# Exploring the causal role of gut microbiota and inflammatory proteins in neuromyelitis optica spectrum disorder: A Mendelian randomization study with mediation analysis

**DOI:** 10.1097/MD.0000000000043967

**Published:** 2025-10-03

**Authors:** Ziqian Yin, Youjia Qiu, Yayi Yang, Sijia Yue, Zhouqing Chen, Jiang Wu

**Affiliations:** a Department of Neurosurgery & Brain and Nerve Research Laboratory, The First Affiliated Hospital of Soochow University, Suzhou, Jiangsu Province, China; b Suzhou Medical College of Soochow University, Suzhou, Jiangsu Province, China; c Department of Gastroenterology, The First Affiliated Hospital of Soochow University, Suzhou, Jiangsu Province, China.

**Keywords:** autoimmune disease, gut microbiota, inflammatory proteins, Mendelian randomization, neuromyelitis optica spectrum disorder

## Abstract

Observational studies highlighted a strong association between gut microbiota (GM) and the onset of neuromyelitis optica spectrum disorder (NMOSD), however, the causality remains unclear. This study investigated the causal relationship between them through two-sample Mendelian randomization (MR) based on genome-wide association studies (GWAS). Two-step MR was used to investigate the potential role of inflammatory cytokines between the causal relationship. Several sensitive analysis were performed to validate the robustness of MR. The MR analysis revealed phylum Tenericutes (*P* = .0357); class Mollicutes (*P* = .0357); genus Eubacterium rectale group (*P* = .0487); genus Barnesiella (*P* = .03); genus Eubacterium xylanophilum group (*P* = .037); and genus Ruminococcus torques group (*P* = .0179) were positively associated with the risk of NMOSD, family Clostridiales vadin BB60 group (*P* = .0244); genus Eggerthella (*P* = .0214); and genus Intestinibacter (*P* = .0308) were negatively correlated with NMOSD. Among 41 inflammatory cytokines, MR showed significant causal effects of Macrophage colony-stimulating factor 1 levels (*P* = .00256), C-X-C motif chemokine 11 levels (*P* = .0478), SIR2-like protein 2 levels (*P* = .00338) and Tumor necrosis factor receptor superfamily member 9 levels (*P* = .0234) on NMOSD. Sensitivity analyses confirmed the robustness of these findings. However, mediated MR analysis did not find evidence supporting inflammatory proteins as mediators in the GM-NMOSD pathway. The study presents evidence for a causal relationship between GM, inflammatory proteins, and NMOSD. Notably, inflammatory proteins do not mediate the pathway from GM to NMOSD, contributing to a deeper understanding of the interplay between GM and NMOSD.

## 1. Introduction

Neuromyelitis optica spectrum disorder (NMOSD) is a chronic autoimmune inflammatory disease of the central nervous system (CNS) caused by demyelinating lesions mainly in optical nerves and spinal cord, which may result in severe motor and sensory deficits.^[1,2]^ It has an estimated worldwide prevalence of 0.5 to 4.4 cases per 100,000 people.^[3]^ The course of NMOSD is repetitive and poses a significant economic burden to patients, especially female patients.^[1,4]^ Notably, serum immunoglobulin G (IgG) antibodies against astrocyte aquaporin 4 (AQP4) have been found in 60% to 90% patients with NMOSD.^[5]^ AQP4-Ab binds to AQP4 expressed on the blood-brain barrier (BBB) astrocytes and causes complement-dependent cellular toxicity and infiltration of neutrophils, eosinophils, and cytokines.^[6]^ Disruption of the BBB leads to oligodendrocyte death, loss of myelin texture, and neuronal damage.^[7]^ Despite the aforementioned potential mechanisms, the initial pathophysiologic triggers that lead to the onset of the disease and result in antibody production remain controversial.

Gut microbiota (GM) is the largest known commensal microbial community in the human body that comprises viruses, fungi, bacteria, and protozoa,^[8]^ and contains 4 trillion microorganisms^[9]^ and 150,000 microbial genomes.^[10]^ As the level of sequencing technologies and research progresses, a deeper understanding of the gut flora has opened up new perspectives for exploring disease mechanisms.^[11]^ Recently, emerging evidence suggests an essential role of GM in the progression of degenerative neurological disorders through the GM–brain axis.^[12,13]^ NMOSD patients were more likely to have antibody responses against H. pylori and gastrointestinal antigens, according to Long et al.^[14,15]^ Furthermore, there is an observed increase in the abundance of *Streptococcus, Aristobacter* spp., *Haemophilus, Veronica, Aeromonas butyricola*, and *Rhodococcus* in patients with NMOSD.^[16,17]^ Intestinal mucosal barrier and GM dysfunction may be due to an imbalance in the host immune system.^[18–20]^ Moreover, undigested food, toxins, microbial products, pro-inflammatory cells, and cytokines may disrupt the intestinal mucosal barrier, allowing pathogens to enter the circulation, disrupting the BBB and inducing an immune response. As a result of disruption of the BBB, these pathogens migrate to the CNS, causing demyelination, axonal loss, and damage to other tissues in the CNS.^[21,22]^ However, GMs may contribute to the development of NMOSD through the AQP4-related pathway. Study conducted by Cree et al found that The AQP4 amino acids 63 to 76 contain 90% homology with amino acids 207 to 216 within the *Clostridium perfringens* adenosine triphosphate-binding cassette transporter protein permease.^[23,24]^ Additionally, there was an increased level of interleukin (IL)-1β, IL-6, and IL-17 produced by CD4 (+) T-helper cells in peripheral blood mononuclear cells in patients with optic neuromyelitis stimulated by *E. coli*. They were positive for these levels, which correlated with the patients’ disability scores.^[25]^ Recent studies have separately explored the possible relationship between some GM and the development of NMOSD. However, there is still a need for a comprehensive study that explores this relationship.

By using the genome-wide association studies (GWAS) summary statistics, Mendelian randomization (MR) can identify causal relationships between exposures and outcomes by utilizing genetic variability as instrumental variables (IVs).^[26]^ MR studies can avoid reverse causation and confounding factors that exist in the vast majority of traditional observational studies.^[27]^ In this study, we comprehensively assessed the possible causal associations of gut microbiomes, inflammatory proteins, immune cells and NMOSD, and explored whether the mediating effects of inflammatory proteins and immune cells in the pathways between gut microbiomes and NMOSD to better understand the preventive and therapeutic potential of GM in NMOSD.

## 2. Materials and methods

### 2.1. Ethics statement

No ethical approval was required for the present study because all data were based on an online-published GWAS analysis that was publicly available. Approval for all of these studies was obtained from the relevant institutional review committees.

### 2.2. Study design

**Figure 1.**
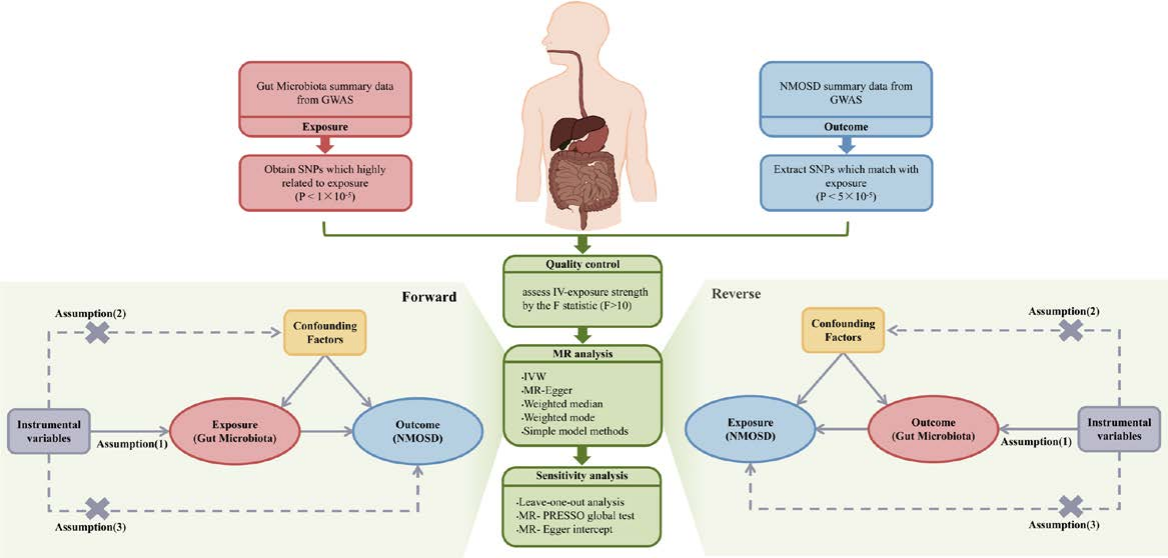
Workflow showing our two-sample bidirectional Mendelian randomization (MR) analysis. GWAS: genome-wide association study; IVs: instrumental variables; IVW: inverse variance weighted; MR-PRESSO: Mendelian randomization pleiotropy residual sum and outlier; SNPs: single nucleotide polymorphisms.

We strictly follow the guidelines outlined in “STROBE-MR” (Strengthening the Reporting of Observational Studies in Epidemiology-Mendelian Randomization). Bidirectional two-sample MR was performed to elucidate the causal association between the GM, inflammatory proteins and NMOSD. Then exploring mediating effects through mediated Mendelian analyses. Figure 1 shows the overview of study design. Three fundamental assumptions must be satisfied in MR analysis: the IVs exhibit a robust association with the exposure factors; No correlation between confounding variables and IVs; IVs can affect outcomes only through exposure factors.^[28]^

### 2.3. Data sources

Genetic variations within the GM were derived from the comprehensive GWAS conducted by the MiBioGen consortium. This consortium boasts the largest collection of published research on gut microbiome genetics to date.^[29]^ The study meticulously procured gene sequencing data from a substantial cohort of 18,340 individuals, encompassing data from 24 distinct cohort studies across 11 countries globally. To standardize the datasets, each was streamlined to a uniform 10,000 reads per sample and subsequently classified using direct classification bins. The study’s inclusion criteria were stringent, requiring a minimum cohort size of 3 and a valid sample size exceeding 3000 individuals. Within each cohort, binary trait locus mapping (mbBTL) analyses were conducted, encompassing a comprehensive scope of 196 taxa and 122,110 variant loci.^[29]^ The genetic data for 91 inflammatory proteins came from the previously GWAS.^[30,31]^

The NMOSD data for this study were obtained from the GWAS catalog website dataset (https://www.ebi.ac.uk/). The data included 215 patients diagnosed with NMOSD, of whom 132 were aquaporin 4 antibody-positive, and a control group with a sample size of 1244.^[32]^ NMOSD patients in this study were diagnosed based on the 2006 diagnostic criteria, which includes transverse myelitis, optic neuritis, and any 2 of the following 3 conditions: longitudinally extensive lesions; brain magnetic resonance imaging inconsistent with multiple sclerosis; seropositive for AQP4-IgG antibody.^[33]^

Inflammatory proteins data were extracted from the EBI GWAS Catalog, with accession numbers spanning from GCST90274758 to GCST90274848 (https://www.ebi.ac.uk/gwas/home). This dataset was inclusive of 11 well-defined cohorts and comprised a substantial participant pool of 14,824 individuals. Genome-wide genetic data for 91 plasma proteins were analyzed utilizing the Olink Target-96 Inflammation panel.^[30]^

### 2.4. IVs selection

Given the scarcity of SNPs identified under the stringent genome-wide significance threshold of *P* < 1 × 10^−8^, we opted for a more lenient locus-wide significance threshold of *P* < 1 × 10^−5^ to procure a broader array of SNPs to serve as IVs,^[34]^ and an aggregation procedure with a strict threshold (*r*^2^ < 0.001, kb = 10,000) was performed to ensure IV independence. In instances where linkage disequilibrium (LD) was detected (*r*^2^ > 0.001), we prioritized the selection of the SNP exhibiting the most significant *P*-value when confronted with high LD.^[35]^ In addition, palindromic SNPs were also removed. To avoid the potential of weak instrumental bias, we calculated F-statistic value using the following formula (F = beta^2^/se^2^), with a value > 10 indicating sufficient strength.^[27]^

### 2.5. MR analysis

Two-sample MR approach was utilized to assess the causal association between GM and NMOSD. The primary analytic method employed was the inverse variance weighted (IVW) method, which amalgamates individual SNP-specific Wald estimates (obtained by dividing the SNP effect size (β) for the outcome by the SNP effect size (β) for exposure) and synthesizes these to provide a comprehensive assessment of the exposure’s impact on the SNP. Fixed or random effects models were selected based on the presence of heterogeneity. When horizontal pleiotropy is absent, IVW prevents the confounders’ effects and achieves unbiased estimation.^[36]^ The odds ratio (OR) reflects the causal link between GM and NMOSD, correlating an increased risk of NMOSD with per SD increase in GM levels. The *P*-value underwent adjustment based on the false discovery rate (FDR) to mitigate the impact of multiple comparisons on the study’s results. We also applied other methods such as MR-Egger, weighted median, weighted mode, and MR polytomous residual sums and outliers (MR-PRESSO), to assess the robustness of MR findings, and the consistent direction of all methods indicated high confidence in the evidence.^[37]^ MR-Egger regression test is a meta-regression of SNP exposure associations with SNP outcome associations with a non-fixed y-axis intercept. This method detects and corrects for directional pleiotropy, but the results may be affected by peripheral genetic variables.^[36,38]^ The weighted median method produces reliable causality estimates when there is a 50% invalid IV.^[39]^ Compared with MR-Egger regression approach, it demonstrates enhanced causality detection, reduced deviation, and a diminished likelihood of type I error, when the instrument strength is not directly influenced by the effect of interest (InSIDE).^[40]^ The MR-PRESSO method employs global testing to identify horizontal pleiotropy, scrutinizing whether the causality estimates are influenced by the exclusion of peripheral SNPs (*P *< .05).^[41]^

### 2.6. Sensitivity analysis

Sensitivity analysis was conducted to detect the robustness of the MR estimates. Heterogeneity among genetic variants was assessed by Cochran Q test and *I*^2^ statistics. MR-PRESSO was utilized to determine potential outlier SNPs, while directional pleiotropy was estimated using the MR-Egger intercept method.^[42]^ The scatter plots depicted the association between BC and meningioma, and the slope represents estimated positive or negative causal effects. Forest plots present causal estimates obtained from each genetic variant, visualizing the heterogeneity of MR results. A funnel plot depicted the distribution of SNPs, and a symmetric distribution indicates no horizontal pleiotropy. Leave-one-out plots were performed multiple times of MR analysis to test whether individual outliers strongly drove causal effects by excluding 1 SNP in turn. In addition, reverse MR was conducted to rule out potential reverse causality.

### 2.7. Intermediary effect

Two-step MR approach was utilized for intermediation analysis, through which the total effect could be divided into direct and indirect effects.^[43]^ The impact of GMs on NMOSD after accounting for potential mediators is denoted as the direct effect; while the intermediary effect from potential mediators is considered indirect effect. We considered the existence of a potential mediator when the following conditions were met: GM was correlated with the inflammatory proteins (β1); inflammatory protein was correlated with NMOSD (β2); and GM was correlated with NMOSD, with no requirement of adjusting mediators (β3). The percentage of mediation was calculated with the ‘Product of coefficient’ by applying the following formula: (β1 × β2)/(β3). All the analyses were conducted using MendelR package (7.8.0) in R software (version 4.3.1).

## 3. Results

### 3.1. Selection of IVs

At the locus-wide significance threshold, a comprehensive analysis identified 2559 SNPs as the definitive IVs for 196 GMs, with a stringent *P*-value threshold of less than 1 × 10^−5^. The distribution of these SNPs across different taxonomic levels was as follows: 9 were associated with phylum, 16 with class, 20 with order, 119 with genera, and 32 with family. For NMOSD patients, we found 229 SNPs with 24 GMs associated with all NMOSD patients. Additionally, all F-statistics exceeded 10, indicating no weak IV bias. Detailed information of gut microbiota and selected IVs are shown in Tables S1 and S2 (Supplemental Digital Content, https://links.lww.com/MD/Q181).

### 3.2. MR analysis

**Figure 2.**
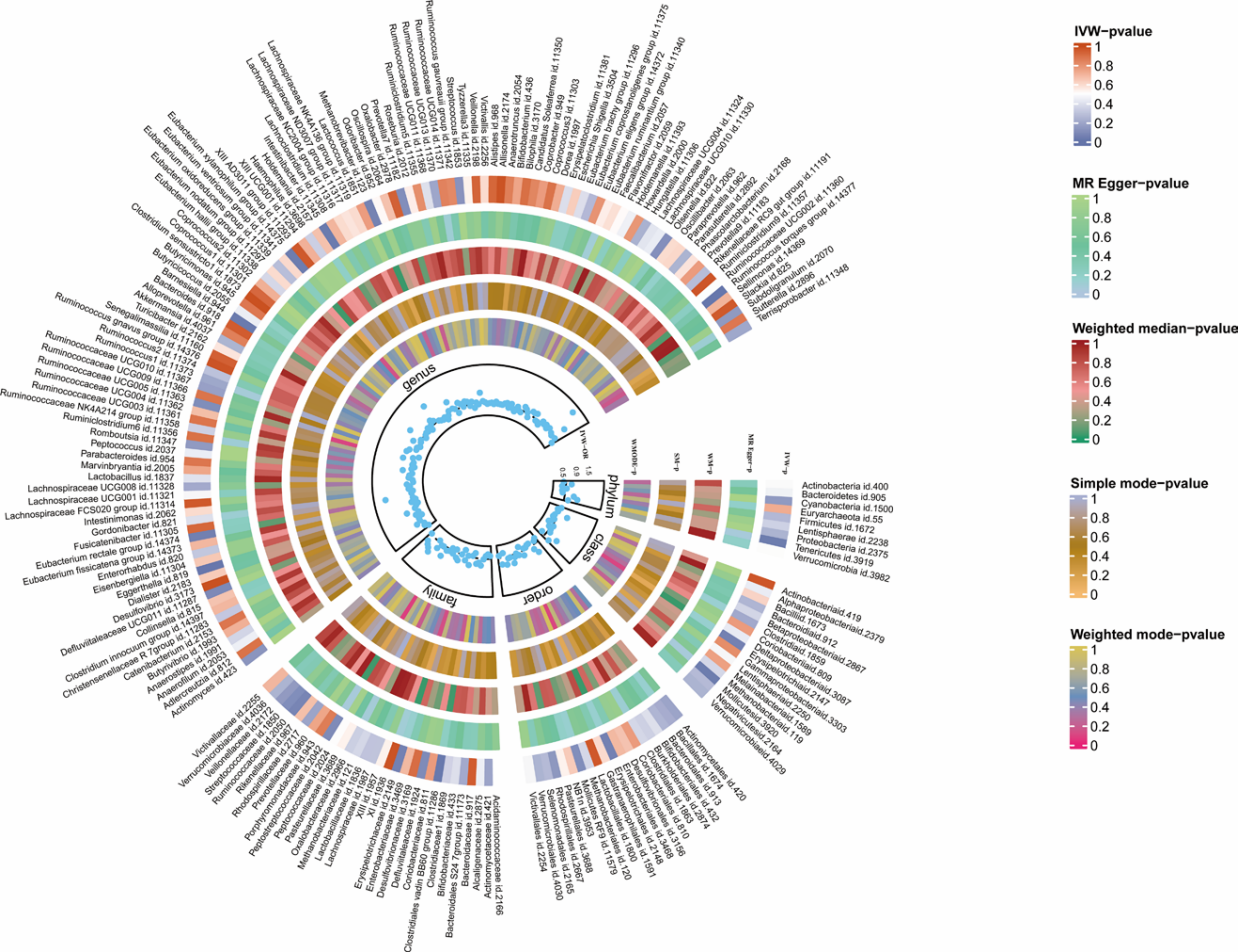
Heatmap plot of the relationship between the gut microbiomes and NMOSD. NMOSD = neuromyelitis optica spectrum disorder.

Heatmap plot demonstrated the results based on the MR analysis of 196 GM and NMOSD (Fig. 2). Detailed analysis information was shown in Table S3 (Supplemental Digital Content, https://links.lww.com/MD/Q181) and Table 1. Using IVW approach, genetically predicted phylum *Tenericutes* (OR = 2.73, 95% CI = 1.07–6.99, *P *= .0357); class *Mollicutes* (OR = 2.73, 95% CI = 1.07–6.99, *P *= .0357); genus *Eubacterium rectale* group (OR = 4.47, 95% CI = 1.01–19.86, *P *= .0487); genus *Barnesiella* (OR = 2.95, 95% CI = 1.09–7.98, *P *= .03); genus *Eubacterium xylanophilum group* (OR = 3.66, 95% CI = 1.08–12.41, *P *= .037); and genus *Ruminococcus torques* group (OR = 5.05, 95% CI = 1.32–19.31, *P *= .0179) were positively associated with the risk of NMOSD; while genetically predicted family *Clostridiales vadin BB60 group* (OR = 0.38, 95% CI = 0.16–0.88, *P *= .0244); genus *Eggerthella* (OR = 0.41, 95% CI = 0.19–0.88, *P *= .0214); and genus *Intestinibacter* (OR = 0.35, 95% CI = 0.13–0.91, *P *= .0308) were negatively correlated with NMOSD risk (Table 1). Supplementary methods such as weight median, weight mode, and MR-Egger also showed the same directions, indicating the robustness of the MR results.

**Table 1:** Summary results of MR (target GM on NMOSD).

Taxa	nsnp	MR analysis				Heterogeneity				Pleiotropy		MR-PRESSO	
		MR-Egger		IVW		MR-Egger		IVW		MR-Egger		Global test	
		OR (95% CI)	*P*-value	OR (95% CI)	*P*-value	Cochran Q	*P*-value	Cochran Q	*P*-value	Egger intercept	*P*-value	RSSobs	*P*-value
Gut microbiota abundance (class Mollicutes id.3920)	12	29.01 (1.56, 537.93)	.047349818	2.73 (1.07, 6.99)	.035678213	6.894275278	.73539	9.69586683	.55793	−0.223424473	.12511	11.9479	.54167
Gut microbiota abundance (family Clostridiales vadin BB60 group id.11286)	14	0.21 (0.02,2.20)	.2164866	0.38 (0.16,0.88)	.024437969	9.476005006	.66182	9.752735457	.71402	0.056775129	.60844	11.1548	.735
Gut microbiota abundance (genus Eggerthella id.819)	10	3.99 (0.13,124.97)	.453391339	0.41 (0.19,0.88)	.021375443	3.410768178	.906	5.161044527	.82005	0.056775129	.60844	6.28038	.83633
Gut microbiota abundance (genus Eubacterium rectale group id.14374)	8	0.03 (0.00,6.00)	.24412496	4.47 (1.01,19.86)	.048731462	2.051985676	.91486	5.777528075	.56595	0.324688325	.10182	7.65044	.58233
Gut microbiota abundance (genus Barnesiella id.944)	14	2.21 (0.08,62.35)	.650809563	2.95 (1.09,7.98)	.033031099	7.141866572	.8481	7.17374496	.89298	0.024370153	.86127	8.32371	.904
Gut microbiota abundance (genus Eubacterium xylanophilum group id.14375)	9	1.53 (0.03,83.82)	.839857522	3.66 (1.08,12.41)	.037048576	4.475985902	.72361	4.676411546	.79154	0.071054185	.6679	5.85585	.80733
Gut microbiota abundance (genus Intestinibacter id.11345)	14	1.23 (0.06,26.50)	.898688623	0.35 (0.13,0.91)	.030791944	8.754619238	.72373	9.466684906	.73685	−0.104918179	.41526	10.9444	.75933
Gut microbiota abundance (genus Ruminococcus torques group id.14377)	9	6.43 (0.16,262.48)	.357943225	5.05 (1.32,19.31)	.017903396	2.883760422	.89554	2.902548715	.94032	−0.01674617	.89483	3.63068	.94933
Gut microbiota abundance (phylum Tenericutes id.3919)	12	29.01 (1.56,537.93)	.047349818	2.73 (1.07,6.99)	.035678213	6.894275278	.73539	9.69586683	.55793	−0.223424473	.12511	11.9479	.564

CI = confidence interval, GM = gut microbiota, IVW = inverse variance weighted, MR = Mendelian randomization, NMOSD = neuromyelitis optica spectrum disorder, NSNPs = number of single nucleotide polymorphisms, OR = odds ratio.

### 3.3. Sensitivity analysis

**Figure 3.**
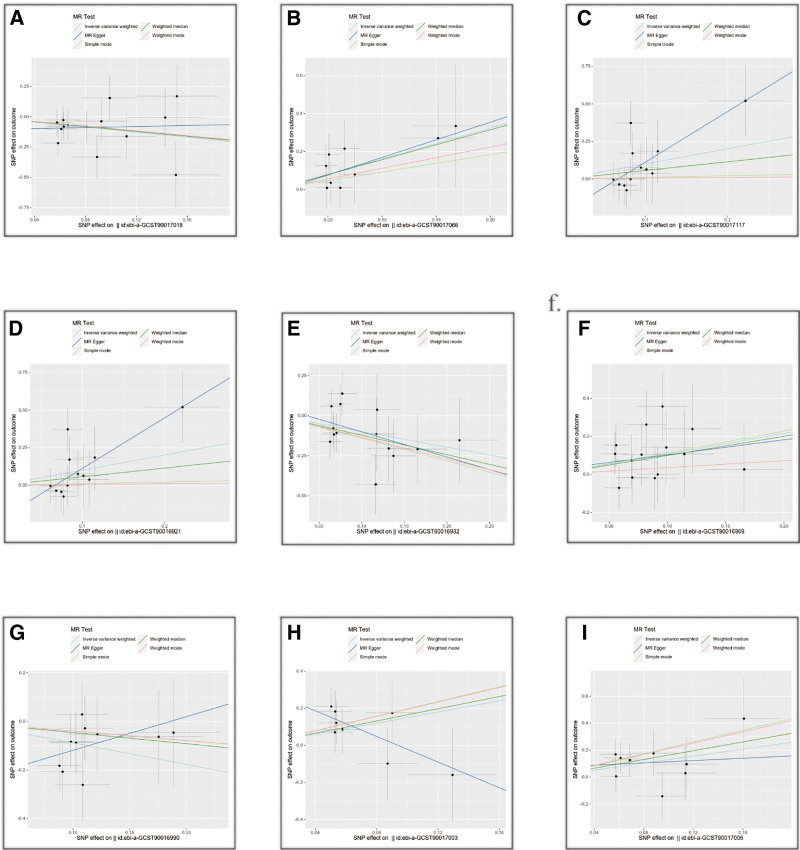
Scatter plots of significant causality of the GM and NMOSD. Scatter plot of the effect size and 95% confidence interval of each SNP on GM and NMOSD risk. The horizontal axis reflects genetic effect of each SNP on GM. The vertical axis represents the genetic effect of each SNP on NMOSD risk. (A) Genus Intestinibacter; (B) genus Ruminococcus torques; (C) phylum Tenericutes; (D) class Mollicutes; (E) family Clostridiales vadin BB60; (F) genus Barnesiella; (G) genus Eggerthella; (H) genus Eubacterium rectale group; (I) genus Eubacterium xylanophilum. GM = gut microbiota, NMOSD = neuromyelitis optica spectrum disorder.

Series of sensitivity analyses were conducted to see if the results were robust when more than 4 SNPs were used as IVs. After completing all sensitivity analyses, we found that the results obtained were robust (Fig. 3 and Tables S4 and S5, Supplemental Digital Content, https://links.lww.com/MD/Q181). In addition, MR-Egger intercept test and MR-PROSSO global test reported no pleiotropy in MR estimates (*P* > .05, Table 1). There was no observed heterogeneity between the selected IVs and NMOSDs based on results of the Cochran Q test (*P *> .05, Table 1). In the leave-one-out analysis, we found that none of the risk estimates between NMOSD and specific bacterial taxa were due to a single SNP. The results were still valid after the exclusion of a more influential SNP (Figs. S1–S9, Supplemental Digital Content, https://links.lww.com/MD/Q179). None of the GMs passed the FDR correction in multiple comparisons. We then further investigated the reverse causality using subtypes of NMOSD as exposure and significant GMs as outcomes. After removing LD, SNPs associated with NMOSD, were obtained from the GWAS database, with each SNP having an F-statistic > 10. The results were shown in Table S6 (Supplemental Digital Content, https://links.lww.com/MD/Q181), no reverse causality was observed between NMOSD and GMs (*P* > .05).

### 3.4. Bi‑directional causal effects of inflammatory proteins and NMOSD

We performed MR analysis of inflammatory proteins with NMOSD, and detailed results are shown in Table S7 (Supplemental Digital Content, https://links.lww.com/MD/Q181). The results show significant causal effects of Macrophage colony-stimulating factor 1 levels (OR = 4.20, 95% CI = 1.65–10.67, *P* = .00256), C-X-C motif chemokine 11 levels (OR = 1.78, 95% CI = 1.01–3.16, *P* = .0478), SIR2-like protein 2 levels (OR = 5.17, 95% CI = 1.72–15.52, *P* = .00338) and Tumor necrosis factor receptor superfamily member 9 levels (OR = 2.30, 95% CI = 1.12–4.71, *P* = .0234) on NMOSD. There was no evidence of heterogeneity or pleiotropy. Detailed heterogeneity and pleiotropy results are available in Tables S8 to S10 (Supplemental Digital Content, https://links.lww.com/MD/Q181).

### 3.5. Mediation analysis

Based on the requirement of mediating role, we performed MR analysis of GM with significant results versus inflammatory proteins with significant results, as detailed in Tables S4 and S8 (Supplemental Digital Content, https://links.lww.com/MD/Q181). and finally 4 inflammatory proteins have the potential to mediate roles in gut flora and NMOSD. We then selected the GM, inflammatory proteins that were significantly associated with NMOSD for MR analysis, and obtained the parts where GM was significantly associated with inflammatory proteins, respectively. The results of further mediating MR analyses, as shown in Table S11 (Supplemental Digital Content, https://links.lww.com/MD/Q181) suggest that there is no mediating role of the GM and NMOSD pathways among the known inflammatory proteins.

## 4. Discussion

This MR analysis is the first to investigate the causal relationship between the GM, inflammatory proteins and NMOSD, and reported an increased relative abundance of genes in the specific genera of GM was associated with a lower risk of NMOSD patients, i.e., phylum *Tenericutes*, class *Mollicutes*, genus *Eubacterium rectale* group, genus *Barnesiella*, genus *Eubacterium xylanophilum* group, and genus *Ruminococcus torques* group were positively associated with the risk of NMOSD. Family *Clostridiales vadin BB60* group, genus *Eggerthella*, and genus *Intestinibacter* were negatively related to the risk of NMOSD.

In recent years, the influence of gut microorganisms on the pathophysiological mechanisms of the gut–brain axis has been emphasized, and it has been found that gut microorganisms influence the development of neuroendocrine and immune-related diseases through the intestinal barrier and the immune system.^[44]^ The structural and functional integrity of the intestinal barrier influences the regulation of intestinal homeostasis, and any damage, including the GMs or drugs can lead to disruption of intestinal homeostasis, which in turn triggers a series of inflammatory responses that disrupt the intestinal barrier.^[45,46]^ When the mucosal barrier is disrupted, potential pathogens such as microorganisms or endotoxins come into direct contact with immune cells such as T and B-lymphocytes, plasma cells, mast cells, and macrophages in the lamina propria. B-lymphocytes receive stimulation from bacterial or viral antigens, and metabolites and are converted to plasma cells, thereby producing and releasing immunoglobulins.^[47]^ An increased percentage of CD38- and CD138-positive cells (plasma cells) in patients with NMOSD was found in a study by Cui et al.^[16,48]^ In addition, the number of CD68-positive cells (macrophages) increased, suggesting that macrophages also play a role in phagocytosis and antigen presentation.^[49]^ Studies have proved that mast cells can release a large number of pro-inflammatory molecules that can damage the tight junction and induce a systemic pro-inflammatory immune response.^[50–53]^ With the disruption of mucosal barriers, pathogens can escape from the intestinal sites and survive at extraintestinal sites.^[54,55]^ Because of the breached barrier, pathogens are transferred to the circulatory system, wherein they induce a chronic or acute inflammatory response increasing host susceptibility to various types of disease.^[45]^ Prolonged presence of molecules and bacteria of intestinal origin in the vicinity of the BBB may compromise the its integrity and thus lead to its collapse.^[16,56]^ Additionally, Cui et al observed a decreased level of 3 types of gap junction proteins (zonula occludens-1, occludin, and claudin-1) in patients with NMOSD, which may result in abnormal intestinal mucosal barrier permeability.^[16,57,58]^ TEM data also showed that cellular gaps were significantly more expansive in these patients, possibly related to a reduction in gap junction proteins.^[16]^ Several studies have shown that most patients with NMOSD have high BBB permeability,^[59,60]^ suggesting that the damage to the gut barrier simultaneously disrupts the integrity and permeability of the BBB.^[46,61,62]^

GMs play a pivotal role in the progression of NMOSD. Gong et al found that patients with NMOSD had more *Streptococcus spp*. as fecal microorganisms than healthy subjects.^[17]^ *Streptococcus spp.* is a part of the normal intestinal flora of the human body and are classified into different species, of which many may also be present in sterile sites causing invasive infections such as bacterial endocarditis, pneumonia, and meningitis.^[63–65]^ A study by Cui et al found that in addition to *Streptococcus*, patients with NMOSD are also enriched with other pro-inflammatory bacteria such as *Granulocystis* spp., *Aspergillus* spp., and *Vibrio desulfuricans* that may also be involved in this pathologic process and play a role in the inflammatory process.^[16]^ Tenericutes (*Eubacterium flexneri*) belongs to one of the placental-specific flora,^[66]^ and Li et al showed that Tenericutes abundance was positively correlated with IL-6 and TNF-α,^[67]^ and that these molecules not only damage the tight junction, but also induce a systemic pro-inflammatory immune response.^[53]^ *Eubacterium rectale group* belongs to the genus *Eubacterium*, which is also a part of the core GMs of humans. *Eubacterium* have been recognized for their crucial involvement in maintaining energy balance, regulating colonic movement, modulating the immune system, and mitigating inflammation within the intestine.^[68]^ Similarly, the *Eubacterium rectale group* positively correlated with pro-inflammatory cytokine levels^[69]^ and was linearly associated with radiation-induced intestinal damage.^[70]^ Cree et al showed that AQP4-specific T-cells cross-react with the adenosine triphosphate-binding cassette transporter protein of *Clostridium perfringens*, which shares sequence homology with AQP4.^[23,71]^ In addition, short-chain fatty acids (SCFAs), produced through the fermentation of dietary fiber by gut microorganisms, have the ability to inhibit histone deacetylase activity. This inhibition promotes the development of regulatory T-cells and also supports the proliferation of precursor cells for macrophages. Influencing CD8 + T-cell function by regulating cellular metabolism has led to a paradoxical role for SCFA in NMOSD.^[72,73]^ A positive correlation was previously shown between *Ruminococcus torques* and SCFA levels.^[74]^ Butyric acid is a type of SCFA, and butyric acid stimulates GFR receptors and reduces inflammation through limiting the production of inflammatory proteins such as NF-B, IL-1 and IL-6.^[75,76]^ Current studies have suggested that *Barnesiella* and *Eubacterium rectale* play an essential role in butyric acid production.^[77,78]^ Our study found the abundance of all 3 genera correlates with the development of NMOSD. Additionally, our results suggest that the genus *Eggerthella* is protective against NMOSD. A growing body of evidence collectively emphasizes the association of *Eggerthella* with a variety of autoimmune diseases such as asthma,^[79]^ multiple sclerosis.^[80]^ For example, *Eggerthella lenta* may increase bile acid metabolites and taurodeoxycholic acid in mice. Both of these can activate oncogenic MAPK/ERK pathway leading to intestinal barrier dysfunction in order to achieve oncogenic effects that trigger colorectal cancer.^[81]^ Since the incidence and development of NMOSD may be affected by bile acids and their metabolites,^[82]^ the *genus Eggerthella* may be associated with NMOSD, but the deeper mechanism requires to be further explored. We also found statistically significant differences between class *Mollicutes*, genus *Intestinibacter*,^[83]^ *Clostridiales vadin BB60* and NMOSD, there are fewer previous studies on the association of in these bacteria with the disease. The relevant directional content needs to be explored in further research studies.

There is growing evidence that the GM could be a potential therapeutic target for treating NMOSD. Dietary control, probiotic nutrition, and fecal flora transplantation have shown promise in improving the efficacy of NMOSD treatment while reducing complications.^[84,85]^ Studies by Wu et al showed that fecal flora transplantation can positively influence the structural composition and functionality of the GM by increasing the level of SCFAs. This discovery provides a new direction for treating neuroimmune diseases in humans.^[84]^ Therefore, our research focuses on exploring therapeutic potential of the specific GM involved in NMOSD and their mechanisms of action, which may translate into possible prevention for NMOSD.

This study boasts several strengths. Firstly, contrasting with traditional epidemiological research, our MR analysis is notably resilient to the influence of confounding variables and the risk of reverse causality. Additionally, we implemented rigorous quality control measures and sensitivity analyses to ensure the reliability of our MR estimates. Furthermore, a stringent FDR correction was applied throughout the MR analysis to significantly reduce the potential for type I errors. Lastly, we conducted MR analyses on the relationship between inflammatory proteins and NMOSD, delving into the potential mediating role of these proteins in the connection between genetic markers and the disease. However, some limitations should also be noted. First, since our GWAS summary data on the GM are at the level of class, order, phylum, family, and genus, some specific GM that relates to more subtle levels (e.g., strain level or species) may be missed and overlooked by this analysis. Second, factors such as the host’s genetics, gender, mode of delivery, surrounding environmental factors, medications, diseases, and dietary habits may affect the diversity of the GM,^[86–88]^ which may have had some impact on our findings. Third, all participants in the dataset used in the study were of European ethnicity, limiting the results to other ethnicities.

## 5. Conclusions

This MR study comprehensively assessed the causal relationship between GM, inflammatory proteins, and NMOSD. Specifically, we found no evidence for a mediating effect of inflammatory factors between GM and NMOSD. This research not only enriches the genetic understanding of NMOSD from a novel perspective but also paves the way for innovative approaches to prevention and treatment strategies in the future.

## Acknowledgments

The author would like to acknowledge the participants and investigators of FinnGen study and MiBioGen.

## Author contributions

**Conceptualization:** Ziqian Yin.

**Data curation:** Ziqian Yin.

**Formal analysis:** Ziqian Yin.

**Funding acquisition:** Ziqian Yin.

**Investigation:** Ziqian Yin.

**Methodology:** Ziqian Yin.

**Project administration:** Zhouqing Chen.

**Supervision:** Jiang Wu.

**Validation:** Youjia Qiu, Jiang Wu.

**Visualization:** Youjia Qiu, Yayi Yang, Jiang Wu.

**Writing – original draft:** Ziqian Yin.

**Writing – review & editing:** Youjia Qiu, Sijia Yue, Zhouqing Chen, Jiang Wu.

## Supplementary Material
