# Supplementary material for: Exploring the causal role of gut microbiota and inflammatory proteins in neuromyelitis optica spectrum disorder: A Mendelian randomization study with mediation analysis
Source: Medicine (Baltimore). 2025 Oct 3;104(40):e43967. doi: 10.1097/MD.0000000000043967 (PMC12499806; doi:10.1097/MD.0000000000043967)
Supplement: Supplementary file 2 [file medi-104-e43967-s002.docx]

Figure S1: Leave one out plot of target Gut microbiota abundance (class Mollicutes id.3920) on NMOSD.

All

rs74603314

rs28537087

rs72901605

rs6043847

rs2464826

rs12566890

rs78169027

rs17214486

rs4885016

rs3768491

rs11890098

rs10108398

-0.5

0.0

0.5

1.0

1.5

2.0

MR leave-one-out sensitivity analysis for

'|| id:ebi-a-GCST90016921' on 'outcome'

Figure S2: Leave one out plot of target Gut microbiota abundance (family Clostridiales vadin BB60 group id.11286) on NMOSD.

All

rs17121075

rs2191834

rs7226487

rs34088226

rs118104867

rs66714985

rs6588624

rs13409132

rs989682

rs55682560

rs10904722

rs28691777

rs10517600

rs7725895

-2.0

-1.5

-1.0

-0.5

0.0

MR leave-one-out sensitivity analysis for

'|| id:ebi-a-GCST90016932' on 'outcome'

Figure S3: Leave one out plot of target Gut microbiota abundance (genus Barnesiella id.944) on NMOSD.

All

rs11155559

rs79795328

rs199035

rs60316894

rs12909713

rs113258194

rs76181748

rs62251337

rs72684847

rs2276875

rs77455852

rs35177866

rs2428166

rs13242616

0.0

0.5

1.0

1.5

2.0

MR leave-one-out sensitivity analysis for

' || id:ebi-a-GCST90016969' on 'outcome'

Figure S4: Leave one out plot of target Gut microbiota abundance (genus Eggerthella id.819) on NMOSD.

All

rs3851328

rs4985746

rs112205261

rs76663501

rs13070736

rs2240838

rs2223081

rs6430926

rs67490567

rs1784446

-1.5

-1.0

-0.5

0.0

MR leave-one-out sensitivity analysis for

'|| id:ebi-a-GCST90016990' on 'outcome'

Figure S5: Leave one out plot of target Gut microbiota abundance (genus Eubacterium rectale group id.14374) on NMOSD.

All

rs10797540

rs314726

rs62547233

rs35398954

rs59427698

rs10248854

rs143694765

rs2884897

0

1

2

3

MR leave-one-out sensitivity analysis for

' || id:ebi-a-GCST90017003' on 'outcome'

Figure S6: Leave one out plot of target Gut microbiota abundance (genus Eubacterium xylanophilum group id.14375) on NMOSD.

All

rs2012708

rs17830032

rs10917203

rs2213117

rs13239072

rs75586835

rs10140184

rs112176119

rs1999224

0

1

2

3

MR leave-one-out sensitivity analysis for

' || id:ebi-a-GCST90017006' on 'outcome'

Figure S7: Leave one out plot of target Gut microbiota abundance (genus Intestinibacter id.11345) on NMOSD.

All

rs118030283

rs9348442

rs478972

rs11109097

rs6062862

rs2098844

rs4327025

rs68093214

rs447950

rs16938435

rs2702387

rs62430350

rs6875660

rs893394

-2.0

-1.5

-1.0

-0.5

0.0

MR leave-one-out sensitivity analysis for

' || id:ebi-a-GCST90017018' on 'outcome'

Figure S8: Leave one out plot of target Gut microbiota abundance (genus Ruminococcus torques group id.14377) on NMOSD.

All

rs10967781

rs4073731

rs8141465

rs10904297

rs77034621

rs12434631

rs1475330

rs8080469

rs35866622

0

1

2

3

MR leave-one-out sensitivity analysis for

' || id:ebi-a-GCST90017066' on 'outcome'

Figure S9: Leave one out plot of target Gut microbiota abundance (phylum Tenericutes id.3919) on NMOSD.

All

rs74603314

rs28537087

rs72901605

rs6043847

rs2464826

rs12566890

rs78169027

rs17214486

rs4885016

rs3768491

rs11890098

rs10108398

-0.5

0.0

0.5

1.0

1.5

2.0

MR leave-one-out sensitivity analysis for

' || id:ebi-a-GCST90017117' on 'outcome'
